# An open challenge to advance probabilistic forecasting for dengue epidemics

**DOI:** 10.1073/pnas.1909865116

**Published:** 2019-11-11

**Authors:** Michael A. Johansson, Karyn M. Apfeldorf, Scott Dobson, Jason Devita, Anna L. Buczak, Benjamin Baugher, Linda J. Moniz, Thomas Bagley, Steven M. Babin, Erhan Guven, Teresa K. Yamana, Jeffrey Shaman, Terry Moschou, Nick Lothian, Aaron Lane, Grant Osborne, Gao Jiang, Logan C. Brooks, David C. Farrow, Sangwon Hyun, Ryan J. Tibshirani, Roni Rosenfeld, Justin Lessler, Nicholas G. Reich, Derek A. T. Cummings, Stephen A. Lauer, Sean M. Moore, Hannah E. Clapham, Rachel Lowe, Trevor C. Bailey, Markel García-Díez, Marilia Sá Carvalho, Xavier Rodó, Tridip Sardar, Richard Paul, Evan L. Ray, Krzysztof Sakrejda, Alexandria C. Brown, Xi Meng, Osonde Osoba, Raffaele Vardavas, David Manheim, Melinda Moore, Dhananjai M. Rao, Travis C. Porco, Sarah Ackley, Fengchen Liu, Lee Worden, Matteo Convertino, Yang Liu, Abraham Reddy, Eloy Ortiz, Jorge Rivero, Humberto Brito, Alicia Juarrero, Leah R. Johnson, Robert B. Gramacy, Jeremy M. Cohen, Erin A. Mordecai, Courtney C. Murdock, Jason R. Rohr, Sadie J. Ryan, Anna M. Stewart-Ibarra, Daniel P. Weikel, Antarpreet Jutla, Rakibul Khan, Marissa Poultney, Rita R. Colwell, Brenda Rivera-García, Christopher M. Barker, Jesse E. Bell, Matthew Biggerstaff, David Swerdlow, Luis Mier-y-Teran-Romero, Brett M. Forshey, Juli Trtanj, Jason Asher, Matt Clay, Harold S. Margolis, Andrew M. Hebbeler, Dylan George, Jean-Paul Chretien

**Affiliations:** ^a^Division of Vector-Borne Diseases, Centers for Disease Control and Prevention, San Juan 00920, Puerto Rico;; ^b^Department of Epidemiology, Harvard T. H. Chan School of Public Health, Boston, MA 02115;; ^c^Data Analytics, Areté Associates, Northridge, CA 91324;; ^d^Systems Integration Branch, Johns Hopkins University Applied Physics Laboratory, Laurel, MD 20723;; ^e^Department of Environmental Health Sciences, Mailman School of Public Health, Columbia University, New York, NY 10032;; ^f^Data to Decisions Cooperative Research Center, Kent Town, SA 5067, Australia;; ^g^Heinz College Information System Management, Carnegie Mellon University, Adelaide, SA 5000, Australia;; ^h^School of Computer Science, Carnegie Mellon University, Pittsburgh, PA 15213;; ^i^Department of Statistics, Carnegie Mellon University, Pittsburgh, PA 15213;; ^j^Department of Epidemiology, Johns Hopkins Bloomberg School of Public Health, Baltimore, MD 21205;; ^k^Department of Biostatistics and Epidemiology, School of Public Health and Health Sciences, University of Massachusetts, Amherst, MA 01003;; ^l^Department of Biology, University of Florida, Gainesville, FL 32611;; ^m^Emerging Pathogens Institute, University of Florida, Gainesville, FL 32611;; ^n^Department of Biological Sciences, University of Notre Dame, Notre Dame, IN 46556;; ^o^Eck Institute for Global Health, University of Notre Dame, Notre Dame, IN 46556;; ^p^Hospital for Tropical Diseases, Oxford University Clinical Research Unit, Ho Chi Minh City, Vietnam;; ^q^Department of Infectious Disease Epidemiology, London School of Hygiene & Tropical Medicine, London WC1E 7HT, United Kingdom;; ^r^Climate and Health Program, Barcelona Institute for Global Health, 08003 Barcelona, Spain;; ^s^College of Engineering, Mathematics and Physical Sciences, University of Exeter, Exeter EX4 4QF, United Kingdom;; ^t^Predictia Intelligent Data Solutions, 39005 Santander, Spain;; ^u^Scientific Computation Program, Oswaldo Cruz Foundation, Rio de Janeiro 21040-900, Brazil;; ^v^Catalan Institution for Research and Advanced Studies, 08010 Barcelona, Spain;; ^w^Department of Mathematical Biology, Indian Statistical Institute, Kolkata, India 700108;; ^x^Pasteur Kyoto International Joint Research Unit for Integrative Vaccinomics, 606-8501 Kyoto, Japan;; ^y^Department of Global Health, Centre National de la Recherche Scientifique, 75016 Paris, France;; ^z^Department of Mathematics and Statistics, Mount Holyoke College, South Hadley, MA 01075;; ^aa^RAND Corporation, Santa MonicaCA 90401;; ^bb^Open Philanthropy, San Francisco, CA 94105;; ^cc^Department of Computer Science and Software Engineering, Miami University, Oxford, OH 45056;; ^dd^F. I. Proctor Foundation for Research in Ophthalmology, University of California, San Francisco, CA 94122;; ^ee^Information Science and Technology, Hokkaido University, Sapporo 060-0808, Japan;; ^ff^Division of Environmental Health Sciences, School of Public Health, University of Minnesota, Twin Cities, MN 55455;; ^gg^VectorAnalytica, Washington, DC 20007;; ^hh^Department of Aeronautical Engineering, Universidade de Sao Paolo, Sao Paolo 13566-590, Brazil;; ^ii^Department of Philosophy, University of Miami, Coral Gables, FL 33146;; ^jj^Department of Statistics, Virginia Tech, Blacksburg, VA 24060;; ^kk^Integrative Biology, University of South Florida, Tampa, FL 33620;; ^ll^Department of Biology, Stanford University, Stanford, CA 94305;; ^mm^Infectious Diseases, College of Veterinary Medicine, University of Georgia, Athens, GA 30602;; ^nn^Odum School of Ecology, University of Georgia, Athens, GA 30602;; ^oo^Department of Geography, University of Florida, Gainesville, FL 32608;; ^pp^School of Life Sciences, University of KwaZulu, Natal 3629, South Africa;; ^qq^Department of Medicine, State University of New York Upstate Medical University, Syracuse, NY 13421;; ^rr^Department of Biostatistics, University of Michigan, Ann Arbor, MI 48109;; ^ss^Department of Civil and Environmental Engineering, West Virginia University, Morgantown, WV 26505;; ^tt^Department of Cell Biology and Molecular Genetics, University of Maryland, College Park, MD 20742;; ^uu^Puerto Rico Department of Health, San Juan 00927, Puerto Rico;; ^vv^Department of Pathology, Microbiology, and Immunology, School of Veterinary Medicine, University of California, Davis, CA 95616;; ^ww^Department of Environmental, Agricultural, and Occupational Health, College of Public Health, University of Nebraska Medical Center, Omaha, NE 68198;; ^xx^Influenza Division, Centers for Disease Control and Prevention, Atlanta, GA 30329;; ^yy^Armed Forces Health Surveillance Branch, Department of Defense, Silver Spring, MD 20904;; ^zz^Climate Program Office, National Oceanic and Atmospheric Administration, Silver Spring, MD 20910;; ^aaa^Leidos supporting the Biomedical Advanced Research and Development Authority, Department of Health and Human Services, Washington, DC 20201;; ^bbb^Bureau of Oceans, International Environmental and Scientific Affairs, US Department of State, Washington, DC 20520;; ^ccc^Office of Science and Technology Policy, The White House, Washington, DC 20502;; ^ddd^BNext, In-Q-Tel, Arlington, VA 22201

**Keywords:** forecast, dengue, epidemic, Peru, Puerto Rico

## Abstract

A wide range of research has promised new tools for forecasting infectious disease dynamics, but little of that research is currently being applied in practice, because tools do not address key public health needs, do not produce probabilistic forecasts, have not been evaluated on external data, or do not provide sufficient forecast skill to be useful. We developed an open collaborative forecasting challenge to assess probabilistic forecasts for seasonal epidemics of dengue, a major global public health problem. Sixteen teams used a variety of methods and data to generate forecasts for 3 epidemiological targets (peak incidence, the week of the peak, and total incidence) over 8 dengue seasons in Iquitos, Peru and San Juan, Puerto Rico. Forecast skill was highly variable across teams and targets. While numerous forecasts showed high skill for midseason situational awareness, early season skill was low, and skill was generally lowest for high incidence seasons, those for which forecasts would be most valuable. A comparison of modeling approaches revealed that average forecast skill was lower for models including biologically meaningful data and mechanisms and that both multimodel and multiteam ensemble forecasts consistently outperformed individual model forecasts. Leveraging these insights, data, and the forecasting framework will be critical to improve forecast skill and the application of forecasts in real time for epidemic preparedness and response. Moreover, key components of this project—integration with public health needs, a common forecasting framework, shared and standardized data, and open participation—can help advance infectious disease forecasting beyond dengue.

## Significance

Forecasts routinely provide critical information for dangerous weather events but not yet for epidemics. Researchers develop computational models that can be used for infectious disease forecasting, but forecasts have not been broadly compared or tested. We collaboratively compared forecasts from 16 teams for 8 y of dengue epidemics in Peru and Puerto Rico. The comparison highlighted components that forecasts did well (e.g., situational awareness late in the season) and those that need more work (e.g., early season forecasts). It also identified key facets to improve forecasts, including using multiple model ensemble approaches to improve overall forecast skill. Future infectious disease forecasting work can build on these findings and this framework to improve the skill and utility of forecasts.

**Fig. 1.**
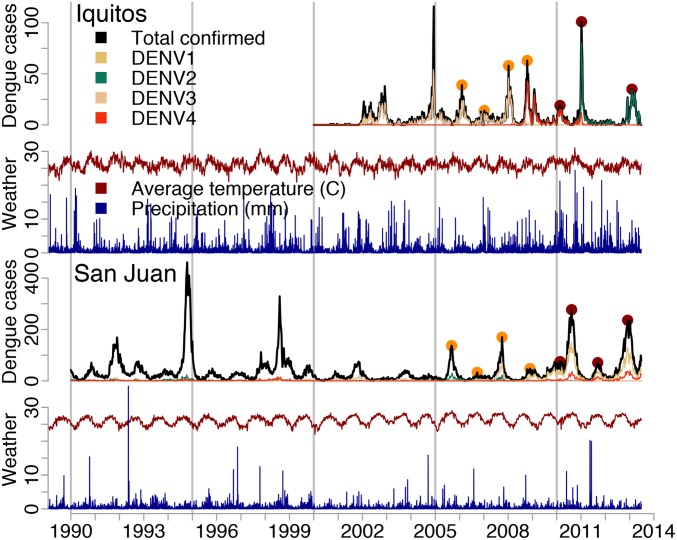
Dengue and climate data for Iquitos, Peru and San Juan, Puerto Rico. The black and colored lines for dengue cases indicate the total and virus-specific weekly number of laboratory-confirmed cases. The yellow and red points indicate the peaks in the training and testing datasets, respectively. The climate data show the weekly rainfall (blue) and mean temperature (red) for Iquitos and San Juan, respectively, from the National Centers for Environmental Prediction Climate Forecast System Reanalysis.

Infectious diseases pose a continuing and dynamic threat globally. The mosquito-transmitted dengue viruses, for example, are endemic throughout the tropical regions of the world and infect millions of people each year (1). In endemic areas, dengue incidence has a clear seasonal pattern but also, exhibits strong interannual variation, with major epidemics occurring every few years (2, 3). In San Juan, Puerto Rico, hundreds of confirmed cases may be reported over an entire interepidemic season, while hundreds of cases can be reported every week during the peak of epidemics (Fig. 1*A*). Timely and effective large-scale interventions are needed to reduce the serious impacts of dengue epidemics on health, healthcare systems, and economies (4, 5). Unfortunately, these epidemics have proven difficult to predict, hindering efforts to prevent and control their impact.

Research on the determinants of dengue epidemics has included both statistical models incorporating historical incidence and climatological determinants (6) and dynamical, mathematical models aimed at identifying both intrinsic and extrinsic drivers (7, 8). This body of research led to important insights, such as the putative influence of various climatological components (9), antibody-dependent enhancement (10, 11), serotype-specific cross-protection (12, 13), and spatial heterogeneity (14) on transmission dynamics.

Despite this substantial body of research, there are currently no operational dengue forecasts with documented prospective forecast skill, and challenges exist for both forecast development and assessment. First, the objectives of published forecasts and outcome metrics vary and are often not tied to specific public health needs. Second, there have been few accessible dengue datasets for forecasting research. Third, differences in data and metrics significantly complicate the comparison of forecasts from different research groups. Fourth, existing evaluations generally assess only point prediction accuracy, ignoring information on forecast confidence. Fifth, evaluations rarely incorporate out-of-sample testing (testing on either reserved or prospective data that were not used to develop and fit the models), the most important test for a forecasting model.

The need to systematically evaluate forecasting tools is widely recognized (15) and motivated multiple US government agencies within the Pandemic Prediction and Forecasting Science and Technology Working Group, coordinated by the White House Office of Science and Technology Policy, to launch an open forecasting challenge in 2015, the Dengue Forecasting Project. First, we worked with epidemiologists from dengue-endemic regions to identify 3 important epidemic forecasting targets: 1) the intensity of the epidemic peak (peak incidence), 2) the timing of that peak (peak week), and 3) the total number of cases expected over the duration of the season (season incidence). Reliable forecasts of these outcomes could improve the allocation of resources for primary prevention (e.g., risk communication, vector control) or secondary prevention (e.g., planning medical staffing, preparing triage units) (16). Additionally, because out-of-sample prediction is an important test of mechanistic causality, forecasts could also provide insight on key drivers of dengue epidemics and therefore, the expected impacts of interventions. Second, we identified 2 dengue-endemic cities, Iquitos, Peru (17, 18) and San Juan, Puerto Rico (19), with serotype-specific incidence data and local climate data that could be released publicly for enough seasons (13 and 23, respectively) to allow training of models and forecasting across multiple seasons (Fig. 1). Third, we established an a priori forecasting framework, including a specific protocol for submitting and evaluating out-of-sample probabilistic forecasts made at 4-wk intervals across 4 training and 4 testing seasons for each of the 3 targets in both locations.

## Results

Sixteen teams submitted binned probabilistic forecasts generated using a variety of approaches, including statistical and mechanistic models and multimodel ensembles (*SI Appendix*, Table S1). All teams used the provided dengue data, 10 (63%) used matched climate data, 2 used serotype data, and 1 used additional data on global climate (e.g., Southern Oscillation Index). Three additional models were developed for comparison: a null model (equal probability assigned to each possible outcome), a baseline statistical time series model (a seasonal autoregressive integrated moving average [SARIMA] model), and a simple ensemble (an average of the probabilities of the 16 team and baseline forecasts).

**Fig. 2.**
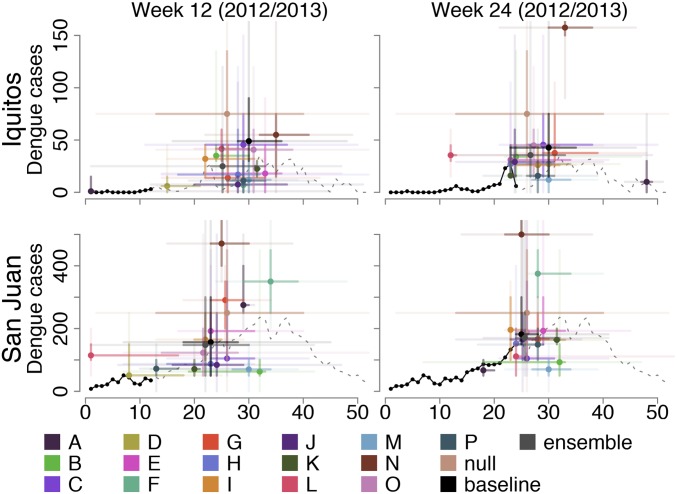
Weeks 12 and 24 forecasts for the 2012/2013 dengue season in Iquitos and San Juan. The solid black lines indicate the most recent data that were available to teams to inform these forecasts, and the dashed lines indicate the data that became available later in the season. The colored points represent point estimates for each team, while the bars represent 50 and 95% prediction intervals (dark and light, respectively). Forecasts for additional time points and seasons as well as for seasonal incidence are shown in *SI Appendix*, Figs. S1 and S2, respectively.

After finalizing models and submitting forecasts for 4 training seasons (2005/2006 to 2008/2009), teams received additional data and had a maximum of 2 wk to submit forecasts for the testing seasons (2009/2010 to 2012/2013). Forecasts varied widely (Fig. 2 and *SI Appendix*, Figs. S1 and S2). For example, forecasts with data up to week 12 and week 24 predicted that the peaks in the 2012/2013 season might have been among the lowest or the highest on record. Confidence also varied: some forecasts were certain of an outcome being in a particular forecast bin, while others had broad 95% prediction intervals spanning the entire range of historical values, and some assigned 0 probability to the observed outcome.

**Fig. 3.**
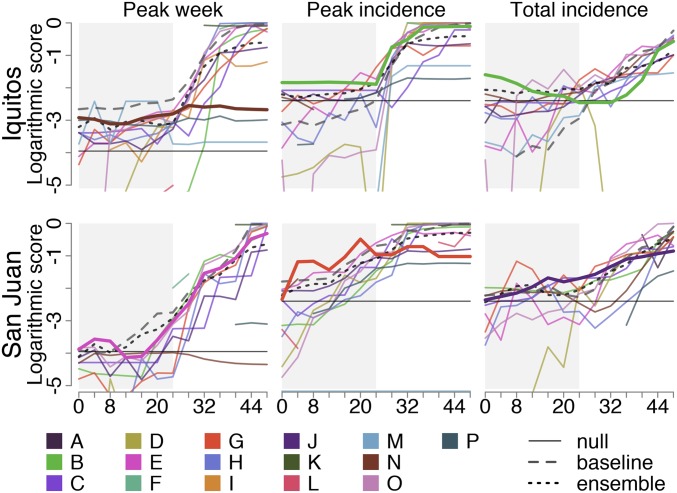
Forecast skill by team, forecast week, and target in the testing seasons (2009/2010 to 2012/2013). Solid colored lines represent the scores of individual teams averaged across all testing seasons for the respective forecast week, target, and location. For each target, the top forecast for the first 24 wk (shaded) is indicated in bold (highest average early season score). The solid black lines indicate the null model (equal probability assigned to all possible outcomes), the dashed gray lines indicate the baseline model, and the dotted black lines indicate the ensemble model. Forecasts with logarithmic scores of less than −5 are not shown. Breaks in lines indicate a score of negative infinity in at least 1 of the testing seasons.

We assessed forecast skill using the logarithmic score, a proper score incorporating probabilistic accuracy and precision. High logarithmic scores indicate consistent assignment of high probability to the eventually observed outcome. Forecast skill increased as seasons progressed for most models (Fig. 3). Some submitted forecasts outperformed both the null and baseline models for early time points, with numerous models showing increased skill around the time of the observed peak (median peak weeks: 22.5 for San Juan and 28 for Iquitos). The peak incidence target for Iquitos in 2011/2012 was not scored, as no distinct peak was identifiable. Forecast calibration (e.g., assigning 70% probability to events that occurred 70% of the time) varied across teams (*SI Appendix*, Fig. S3) and was strongly associated with forecast skill (*SI Appendix*, Fig. S4).

**Fig. 4.**
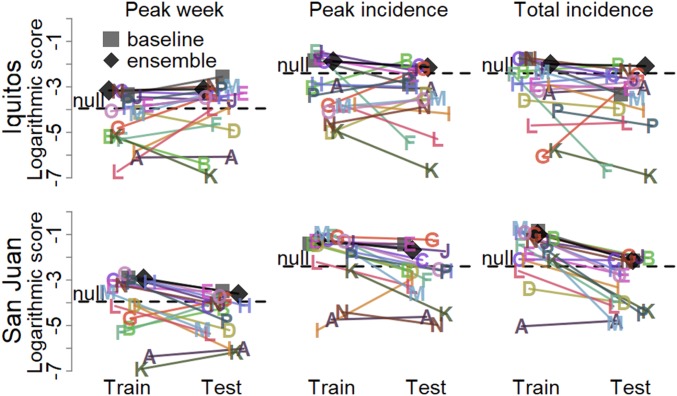
Overall forecast scores for weeks 0 to 24 in the training (2005/2006 to 2008/2009) and testing (2009/2010 to 2012/2013) seasons. Each point is the average target- and location-specific log score for a model in the training (left side; light shading) and testing (right side; dark shading) seasons. The horizontal dispersion within training and testing scores is random to improve visualization. The null forecast for each target is represented by a horizontal line. Numerous forecasts assigned 0 probability to at least 1 observed outcome. Those individual forecast probabilities were changed to 0.001 before calculating the logarithmic scores.

The highest skill early season forecasts (weeks 0 to 24) for each target–location pair were submitted by Team N (University of California, San Francisco, peak week, Iquitos), Team E (VectorBiTE, peak week, San Juan) (20), Team B (Breaking Bad Bone Fever, peak incidence and total incidence, Iquitos) (21), Team G (Areté, peak incidence, San Juan), and Team J (Delphi, total incidence, San Juan) (Figs. 3 and 4 and *SI Appendix*, Table S1). Many teams outscored both the null model and for each target except peak week, the baseline model. The ensemble forecast outperformed most individual models and was the only forecast to outperform the null model for every target. Training season forecasts showed similar patterns of low early season skill and overconfidence by some models, and numerous models outperformed the baseline and null models (*SI Appendix*, Fig. S5).The top teams differed for all targets except peak week in Iquitos (Fig. 4 and *SI Appendix*, Table S2), but the ensemble forecasts outperformed the majority of individual forecasts and the null forecast for all targets for all 8 seasons.

To assess extrinsic factors that may impact forecast skill, we fitted a series of regression models to target-, location-, and season-specific variables (*SI Appendix*). Scores were higher for forecasts made later in the season (0.043 per week, 95% confidence interval [95% CI]: 0.039 to 0.046), seasons with lower peak incidence (0.43 per location-specific SD, 95% CI: 0.37 to 0.49), seasons with earlier peaks (0.048 per week prior to long-term location-specific mean, 95% CI: 0.040 to 0.057), San Juan (0.65, 95% CI: 0.54 to 0.76), and targets with fewer bins (peak and seasonal incidence, 0.0257 per bin, 95% CI: 0.0221 to 0.0293) (*SI Appendix*, Table S3).

Comparing high-level forecasting approaches across all targets and all 8 seasons while controlling for the differences by forecast week, season characteristics, location, and the numbers of bins (described above), we found that logarithmic scores were higher for teams using ensemble approaches (mean difference: 1.02, 95% CI: 0.91 to 1.13) (*SI Appendix*, Table S3). Forecasts from models incorporating mechanistic approaches (e.g., compartmental models or ensemble models with at least 1 mechanistic submodel) had lower logarithmic scores (−0.65, 95% CI: −0.80 to −0.49) than purely statistical approaches. Additionally, models using climate data had lower logarithmic scores (−0.14, 95% CI: −0.19 to −0.09). Relatedly, we found that forecasts using ensemble approaches tended to be better calibrated (−0.0010, 95% CI: −0.0034 to 0.0007) and that those using mechanistic approaches or climate data were less so (*SI Appendix*, Table S4). We did not compare models using serotype data or incorporating vector population dynamics, as only 2 models included serotype data (using them in different ways), and all but 1 mechanistic model included modeled vector populations (actual vector data were not available).

## Discussion

Research aimed at forecasting epidemics and their impact offers tantalizing opportunities to prevent or control infectious diseases. Although many epidemic forecasting tools promise high accuracy, they have largely been fit to specific, nonpublic datasets and assessed only on historical data rather than future, unobserved outcomes. Here, we executed a multimodel assessment of out-of-sample probabilistic forecasts for key seasonal characteristics of dengue epidemics. Comparing these forecasts provides insight on current capabilities to forecast dengue, our understanding of the drivers of dengue epidemics, challenges to forecast skill, and avenues for improvement.

Good forecasts should identify possible outcomes relevant to decision makers and reliably assign probabilities to those outcomes (22). Proper scores (23, 24) of probabilistic forecasts, such as the logarithmic score used here, have distinct advantages over more common point prediction error metrics. Error only measures 1 dimension of forecast skill, the distance between the estimated and observed outcomes, and does not consider confidence, an essential characteristic for stochastic outcomes. Logarithmic scores for the submitted forecasts revealed low early season forecast skill, with many forecasts performing worse than a null forecast that assigned an equal probability to each possible outcome. Even in endemic areas with strong seasonal transmission patterns, epidemics are difficult to predict at time horizons of several months or more.

Nonetheless, several teams consistently outperformed the null model for each target–location pair, indicating that, even in early weeks, models provided some reliable information about what was likely to happen. Not surprisingly, forecasts improved substantially as seasons progressed and data accumulated. As more data are reported, the likely outcomes are reduced, and forecasting is easier (e.g., if 1,100 cases have been reported by week 40, it is impossible that the season total will be less than 1,000 and extremely unlikely that it would exceed 10,000). Despite this, some models had decreased or steady late season skill, possibly indicating that they did not fully account for data updates. Week-to-week incidence varies substantially, making peaks hard to identify in real time, and therefore, models with high midseason to late season skill may be very useful for situational awareness.

Overall scores varied by target, location, and season. Differences in target-specific scores were not associated with target-specific entropy, implying that target-specific differences were more likely due to study design than intrinsic differences in predictability. Specifically, the peak week target had more bins (52 vs. 11), and therefore, probabilities were distributed across more bins, leading to lower probabilities for the outcomes and lower scores. Higher scores for San Juan compared with Iquitos may reflect differences in dynamics, the availability of more historical data, or the location-specific bin selection. This difference was not related to location-specific variability, as target-specific entropy was similar or higher for San Juan (peak week: 1.28 for Iquitos and 2.08 for San Juan; peak incidence: 1.75 and 1.73, respectively; and season incidence: 1.28 and 1.39, respectively). However, the long-term dynamics in the 2 locations were markedly different, with more recent introduction and serotype replacement in Iquitos vs. decades of hyperendemic transmission in San Juan. The effect of these differences and simply the availability of more historical data for San Juan are not distinguishable in this study. Finally, we found that forecast skill was lower for seasons with later and higher peaks. The association with later peaks may indicate a particular challenge of late seasons or a more general association with atypical peak timing rather than a late season per se, or it may simply reflect a higher proportion of forecasts being made before the peak, when there is more uncertainty. Influenza forecasts also tend to perform worse in late seasons (25). The association of low forecast skill with high incidence is also a key challenge; seasonal cycling is generally predictable, but high-incidence epidemics, the biggest challenge for public health, are the hardest to predict.

A wide variety of modeling approaches was used, including different criteria for data selection (e.g. climate data, lags), model frameworks (e.g., mechanistic, statistical), parameter assignment methods (e.g., fitting, specifying), and forecast generation procedures (e.g., model selection, combination). Because there are so many potential options for these components, the 17 models that we evaluated (teams and baseline) only represent a small subspace of all possible models. We, therefore, restricted our analysis to 3 high-level characteristics represented by multiple forecasts (climate data, a mechanistic model, or an ensemble approach), recognizing that even these findings may not be generalizable. Suitable climatic conditions are biologically necessary for dengue virus transmission, yet models including climate data had less skill than models that did not. One challenge is that climate forecasts may be more useful than historical data for dengue forecasts, but climate forecasts have their own uncertainty (26). Moreover, it is possible that better climate forecasts may not improve dengue forecasts. For example, climate may determine dengue seasonality, but models characterizing seasonality using historical dengue data alone (e.g., the baseline SARIMA model) may be able to provide equivalent information about expected future incidence (6). Incorporating additional data also increases model complexity in the form of variability in those data, parameters, and structural assumptions. Including estimated parameters or model structures that better match historical data or biological relationships may come at the expense of lower out-of-sample forecast skill.

Our finding that statistical models generally outperformed mechanistic models is another indicator of the potential downside of overly complex forecasting models. Statistical models may have performed slightly better because robust uncertainty estimates are easier to generate with standard statistical packages compared with tailored mechanistic models. For example, the relatively simple baseline SARIMA models (4 parameters for Iquitos, 5 for San Juan) were developed with a standard statistical package and generally performed well compared with more complex models, including having the best overall calibration and the highest skill forecasts for peak week. Although simple models have also performed well in other forecasting challenges (27, 28), mechanistic models should not be dismissed. Mechanistic models allow for the incorporation of biological interactions (e.g., serotype interaction, spatial heterogeneity) and are essential for estimating the impacts of potential interventions (29). Statistical models can be used to guide development of better mechanistic models, capturing key components of good forecasts, such as seasonality, short-term autocorrelation, and accurate characterization of uncertainty. Moreover, hybrid approaches such as ensemble models, including statistical and mechanistic submodels, may be able to leverage advantages of both approaches.

Ensemble approaches were used by almost half the teams (7 of 16) (20, 21, 30) and on average, had better calibration and higher forecast skill than forecasts generated from single models. Moreover, a simple ensemble of all of the forecasts was among the highest scoring forecasts for every target and time point and was the only forecast to outperform the null forecast for all targets. Despite being a simple average of many forecasts, most of which performed substantially worse on their own, the ensemble balanced uncertainty across competing models with different assumptions and parameters, improving calibration by hedging bets when submodels disagreed and consolidating them when there was agreement. This cross-model modulation of uncertainty leads to higher skill forecasts as seen here and in other challenges (25, 28) and highlights a key advantage of multimodel and multiteam forecasting: a suite of models is likely to outperform any single approach (31). It also points to an important future research area: optimization of ensembles with fitted and dynamic weights.

While these insights can drive future research, there were also key limitations. For example, 2 potentially important dengue drivers were not assessed: vector populations and dengue virus serotypes. Vector data were simply not available on a spatiotemporal scale commensurate with the dengue data used here. Because numerous studies have shown that the interactions between dengue virus serotypes and human immunity may be a critical driver of long-term dengue dynamics (32), we provided datasets, including serotype data. However, only 2 teams chose to use them: one as an indicator of recent introduction of a serotype and the other in a complex 4-strain mechanistic compartmental model. The importance of serotype data for forecasting remains an open and important question, particularly for long-term dengue-endemic areas, such as Southeast Asia, where these effects may be strongest. Datasets with such extensive historical data are rare but offer an opportunity to identify key epidemic drivers that could inform current and future surveillance strategies in areas with less comprehensive historical data. Additionally, the comparison of approaches was only among the limited set used by the teams, not a comprehensive library of approaches. Different data and models have the potential to improve forecasts, but additional evidence is needed to understand which data and relationships are most important for dengue forecasting. Those determinations will also be key to future surveillance strategies, identifying the most important data to capture.

The challenge structure also had some limitations. Forecasts were evaluated on probabilities that were binned according to prespecified bins. Because targets are on different scales, it is not clear how to objectively define these bins to enable between-target comparison. It is also unclear how closely the bins should be tied to very specific decision-making needs, such as identifying an “outbreak,” a concept with a wide variety of definitions that are intrinsically dependent both on surveillance and a threshold selection algorithm (33). Binned forecasts enable more comprehensive comparison of forecasts without selection of a specific threshold and allow scaling to higher levels, such as the binary probability of incidence exceeding a particular threshold. The datasets differed in both amount of data (13 seasons for Iquitos, 23 for San Juan) and characteristics of local dengue (serotype replacement in Iquitos, hyperendemicity in San Juan). Yet, those only represent 2 locations of the many where dengue is endemic. More datasets will be needed to determine the generalizability of forecasting tools, but few datasets with this level of detail exist. To evaluate forecasts over multiple seasons, the project was designed to use retrospective data and therefore, was not truly prospective. To facilitate forecasting at 13 time points per season, some future data were shared. To assure appropriate use, all teams agreed to forecast using data exclusively from weeks prior to and including the forecast week, and testing data were only available for 2 wk and only after selection of a final model and submission of training forecasts. Another challenge posed by these retrospective datasets is that they do not represent real-time reporting with its intrinsic reporting delays, another key forecasting challenge. Short-term forecasts for seasonal influenza show promise at helping bridge this gap (25), but comparable data were not available for this challenge, and the problem is far from solved. The datasets also do not represent all infections or even all cases, as we focused on laboratory-confirmed cases. Some cases do not seek care, do not have access to care, or are misdiagnosed. This may impact forecast model inputs and outputs, as both the underlying transmission dynamics and the case burden are imperfectly captured by data on confirmed cases.

Nonetheless, this project highlights important lessons for the larger panorama of challenges to advance the research and application of epidemic forecasting for public health. First, to make forecasts relevant to decision making in outbreak responses, targets should be clearly and quantitatively defined, and they should directly address specific public health needs. To integrate forecasts into decision making, it will be vital to refine the way that forecasts are communicated and maximize their operational relevance. Second, more participation leads to more information gain both for improved forecast skill via ensembles and also, for characterizing the strengths and weakness of different modeling approaches (25, 28, 34). Opening new data, facilitating access, and presenting engaging problems can drive participation and enable this type of research. Third, forecast skill should be openly evaluated on out-of-sample data with prespecified metrics that consider uncertainty. Self-evaluation of point predictions on data that are not openly accessible does little to characterize the utility of a forecasting tool. Good forecasts should be able to 1) differentiate between possible out-of-sample outcomes and 2) accurately express confidence in those predictions. Together, these components can be the building blocks for future forecasting systems, such as those that have transformed weather and storm forecasting (35).

Dengue remains a major public health challenge, and decades of dengue research have led to little progress in prospective prediction of dengue epidemics. Here, we identified key challenges and established a framework with datasets to help advance this research specifically toward targets that would benefit public health and forecasting science. Next generation models by the participating teams and others should adopt the testing–training framework, data, and metrics to assess forecast performance using the scores of the forecasts published here as benchmarks to measure advances in forecasting skill. At the same time, it may be important to refine targets and identify new targets to maximize public health utility. Additional datasets to retrospectively and prospectively develop and validate forecasts will be critical for demonstrating forecast skill and reliability across multiple seasons (and multiple locations for broader implementation). The recent epidemics of chikungunya and Zika viruses have further complicated clinical and laboratory-based surveillance for dengue and created a more complex immunological landscape for flaviviruses, changes that create new challenges for interpreting surveillance data and forecasting. There is also a need for improved surveillance data systems to ensure that data are machine readable and available in real time to support truly prospective, real-time forecasts. Lastly, better forecasts will drive interventions, increasing the importance of better mechanistic models that can both forecast and estimate the impact of interventions. These are formidable challenges, but through probabilistic forecasting projects, such as the one reported here, the community can move this research forward, translating the research into public health tools that can transform the way that we prepare for and respond to epidemics.

## Materials and Methods

### Data.

Weekly laboratory-confirmed and serotype-specific dengue surveillance data were provided for 2 endemic locations: Iquitos, Peru (17, 36) and San Juan, Puerto Rico (19). Data were time referenced starting with 1 January, and data from 31 December (30 December for leap years) were removed to ensure 52 wk/y. The week with the lowest average incidence over the training period was then selected as the end week for the transmission season (week 26 in Iquitos and week 17 in San Juan) such that each dengue season began on the following week. All data were final, reflecting all cases with onset in each week regardless of reporting delays that affected the availability of data in real time. The data were divided into training data (Iquitos: 2000/2001 to 2008/2009, San Juan: 1990/1991 to 2008/2009) and testing data (2009/2010 to 2012/2013 for both locations). Climate and environmental data were provided for both locations (*SI Appendix*). Complete datasets are available at https://predict.cdc.gov. Participants were permitted to use other data (e.g., social media or demographic data) but not data on dengue in the study locations or nearby locations unless those data were made available to all participants.

### Forecast Targets.

For each season and location, the following targets were forecasted: *Peak week*, the week with the highest incidence of dengue (or undefined if more than 1 wk had the highest number of cases); *Peak incidence*, the number of dengue cases reported in the peak week; and *Total incidence*, the total number of confirmed dengue cases reported over the season.

Each forecast included a point estimate and a binned probability distribution. For peak week, each bin represented a single week (i.e., 1, 2, …, 52). For peak and total incidence, 11 bins were chosen empirically by setting an upper bound ∼50% higher than the maxima observed in the training data. The maximum observed peak incidence in Iquitos was 116 cases, and we used bins of width 15 cases to cover up to 149 with 10 bins plus a final bin for 150 or more cases. For San Juan, with a maximum of 461 cases, we used bins of width 50 and 500 or more as the final bin. For total incidence, the maxima observed were 715 and 6,690 cases for Iquitos and San Juan, respectively. Bin widths were selected at 100 and 1,000 cases, respectively, with the last bin for >1,000 or >10,000 cases. Probabilities between 0 and 1 were assigned to each bin, summing to 1.0 for each specific forecast (e.g., the week 4 forecast for peak week in San Juan 2005/2006).

### Forecasting.

The forecasting project started on 5 June 2015, with public announcement of the challenge and online publication of the training datasets and forecast templates. Forecasting occurred in 2 stages. First, to participate, each team was required to submit a model description and a set of formatted forecasts for all 3 targets at both locations for the last 4 seasons of the training dataset (2005/2006 to 2008/2009) at 13 time points per season (weeks 0, 4, 8, …, 48) by email by 12 August 2015. Each team explicitly stated that these were out-of-sample forecasts using only the data from prior time points in all datasets used. The training forecasts and model descriptions were evaluated for adherence to the guidelines. Teams meeting those guidelines received the testing data on 19 August and had 2 wk to generate and submit forecasts from the same model for the 4 testing seasons (2009/2010 to 2012/2013; deadline: 2 September 2015). The only incentives for participation were the provision of data, the opportunity to compare prospective forecasts, and the opportunity to participate in the development of this manuscript. Details are available at https://dengueforecasting.noaa.gov and https://predict.cdc.gov and in ref. 37.

We analyzed 3 additional models for comparison: a null model, a baseline model, and an ensemble model. The null model assigned equal probabilities to all bins (e.g., 1 of 52 for each possible peak week). The baseline models were SARIMA models, capturing seasonal trends and short-term autocorrelation [SARIMA(1, 0, 0)(4, 1, 0)_12_ for San Juan and SARIMA(1, 0, 0)(3, 1, 0)_12_ for Iquitos] (6). Finally, the ensemble model was created by averaging the probability bins from all team forecasts and the baseline forecast.

### Evaluation.

All forecasts were evaluated using the logarithmic score, a proper scoring rule based on probability densities (24, 38). The logarithmic score is the average logarithm of the probability assigned to the observed outcome bin (described above), *p*_*i*_, over *n* predictions: $S_{n}=\frac{1}{n}\sum_{i=1}^{n}\log\left(p_{i}\right)$. We used Bayesian generalized linear models to identify season and model characteristics potentially related to forecast skill (*SI Appendix*). All analyses were performed in R (https://www.r-project.org/).

## Supplementary Material

Supplementary File
