# Supplementary material for: An open challenge to advance probabilistic forecasting for dengue epidemics
Source: Proc Natl Acad Sci U S A. 2019 Nov 11;116(48):24268–74. doi: 10.1073/pnas.1909865116 (PMC6883829; doi:10.1073/pnas.1909865116)
Supplement: Supplementary File [file pnas.1909865116.sapp.pdf]

Supplementary Information for

An open challenge to advance probabilistic forecasting for dengue epidemics

Authors

Michael A. Johansson[a,b,1], Karyn M. Apfeldorf[c], Scott Dobson[c], Jason Devita[c], Anna L. Buczak[d], Benjamin Baugher[d], Linda J. Moniz[d], Thomas Bagley[d], Steven M. Babin[d], Erhan Guven[d], Teresa K. Yamana[e], Jeffrey Shaman[e], Terry Moschou[f], Nick Lothian[f], Aaron Lane[f], Grant Osborne[f], Gao Jiang[g], Logan C. Brooks[h], David C. Farrow[h], Sangwon Hyun[i], Ryan J. Tibshirani[h,i], Roni Rosenfeld[h], Justin Lessler[j], Nicholas G. Reich[k], Derek A. T. Cummings[l,m], Stephen A. Lauer[k], Sean M. Moore[n,o], Hannah E. Clapham[p], Rachel Lowe[q,r], Trevor C. Bailey[s], Markel García-Díez[t], Marilia Sá Carvalho[u], Xavier Rodó[r], Tridip Sardar[v], Richard Paul[w,x], Evan L. Ray[y], Krzysztof Sakrejda[k], Alexandria C. Brown[k], Xi Meng[k], Osonde Osoba[z], Raffaele Vardavas[z], David Manheim[aa], Melinda Moore[z], Dhananjai M. Rao[bb], Travis C. Porco[cc], Sarah Ackley[cc], Fengchen Liu[cc], Lee Worden[cc], Matteo Convertino[dd], Yang Liu[ee], Abraham Reddy[ee], Eloy Ortiz[ff], Jorge Rivero[ff], Humberto Brito[ff,gg], Alicia Juarrero[ff,hh], Leah R. Johnson[ii], Robert B. Gramacy[jj], Jeremy M. Cohen[jj], Erin A. Mordecai[kk], Courtney C. Murdock[ll,mm], Jason R. Rohr[n,o], Sadie J. Ryan[m,nn,oo], Anna M. Stewart-Ibarra[pp], Daniel P. Weikel[qq], Antarpreet Jutla[rr], Rakibul Khan[rr], Marissa Poultney[rr], Rita R. Colwell[ss], Brenda Rivera-García[tt], Christopher M. Barker[uu], Jesse E. Bell[vv], Matthew Biggerstaff[ww], David Swerdlow[ww], Luis Mier-y-Teran-Romero[a,j], Brett M. Forshey[xx], Juli Trtanj[yy], Jason Asher[zz], Matt Clay[zz], Harold S. Margolis[a], Andrew M. Hebbeler[aaa,bbb], Dylan George[bbb,ccc], Jean-Paul Chretien[bbb,ddd]

Affiliations

a. Division of Vector-Borne Diseases, Centers for Disease Control and Prevention, San Juan, PR 00920
b. Department of Epidemiology, Harvard TH Chan School of Public Health, Boston, MA 02115
c. Data Analytics, Areté Associates, Northridge, CA 91324
d. Systems Integration Branch, Johns Hopkins University Applied Physics Laboratory, Laurel, MD 20723
e. Department of Environmental Health Sciences, Mailman School of Public Health, Columbia University, New York, NY 10032
f. Data to Decisions Cooperative Research Center, Kent Town, South Australia, Australia 5067
g. Heinz College Information System Management, Carnegie Mellon University, Adelaide, South Australia, Australia 5000
h. School of Computer Science, Carnegie Mellon University, Pittsburgh, PA 15213
i. Department of Statistics, Carnegie Mellon University, Pittsburgh, PA 15213

www.pnas.org/cgi/doi/10.1073/pnas.1909865116

j. Department of Epidemiology, Johns Hopkins Bloomberg School of Public Health, Baltimore MD 21205
k. Department of Biostatistics and Epidemiology, School of Public Health and Health Sciences, University of Massachusetts, Amherst, MA 01003
l. Department of Biology, University of Florida, Gainesville, FL 32611
m. Emerging Pathogens Institute, University of Florida, Gainesville, FL 32611
n. Department of Biological Sciences, University of Notre Dame, Notre Dame, IN 46556
o. Eck Institute for Global Health, University of Notre Dame, Notre Dame, IN 46556
p. Hospital for Tropical Diseases, Oxford University Clinical Research Unit, Ho Chi Minh City, Vietnam
q. Department of Infectious Disease Epidemiology, London School of Hygiene & Tropical Medicine, London, United Kingdom WC1E 7HT
r. Climate and Health Program, Barcelona Institute for Global Health, Barcelona, Spain 08003
s. College of Engineering, Mathematics and Physical Sciences, University of Exeter, Exeter, United Kingdom EX4 4QF
t. Predictia Intelligent Data Solutions, Santander, Spain 39005
u. Scientific Computation Program, Oswaldo Cruz Foundation, Rio de Janeiro, Brazil 21040-900
v. Department of Mathematical Biology, Indian Statistical Institute, Kolkata, India 700108
w. Pasteur Kyoto International Joint Research Unit for Integrative Vaccinomics, Kyoto, Japan 606-8501
x. Department of Global Health, Centre National de la Recherche Scientifique, Paris, France 75016
y. Department of Mathematics and Statistics, Mount Holyoke College, South Hadley, MA 01075
z. RAND Corporation, Santa Monica, CA 90401
aa. Open Philanthropy, San Francisco, CA 94105
bb. Department of Computer Science and Software Engineering, Miami University, Oxford, OH 45056
cc. F. I. Proctor Foundation for Research in Ophthalmology, University of California San Francisco, San Francisco, CA 94122
dd. Information Science and Technology, Hokkaido University, Sapporo, Japan 060-0808
ee. Division of Environmental Health Sciences, School of Public Health, University of Minnesota, Twin Cities, MN 55455
ff. VectorAnalytica, Washington, DC 20007
gg. Department of Aeronautical Engineering, Universidade de Sao Paolo, Sao Paolo, Brasil 13566-590
hh. Department of Philosophy, University of Miami, Coral Gables, FL 33146
ii. Department of Statistics, Virginia Tech, Blacksburg, VA 24060
jj. Integrative Biology, University of South Florida, Tampa, FL 33620
kk. Department of Biology, Stanford University, Stanford, CA 94305
ll. Infectious Diseases, College of Veterinary Medicine, University of Georgia, Athens, GA 30602
mm. Odum School of Ecology, University of Georgia, Athens, GA 30602
nn. Department of Geography, University of Florida, Gainesville, FL 32608
oo. School of Life Sciences, University of KwaZulu, Natal, South Africa 3629
pp. Department of Medicine, State University of New York Upstate Medical University, Syracuse, NY 13421
qq. Department of Biostatistics, University of Michigan, Ann Arbor, MI 48109
rr. Department of Civil and Environmental Engineering, West Virginia University, Morgantown, WV 26505
ss. Department of Cell Biology and Molecular Genetics, University of Maryland, College Park, MD 20742
tt. Puerto Rico Department of Health, San Juan, PR 00927
uu. Department of Pathology, Microbiology, and Immunology, School of Veterinary Medicine, University of California, Davis, CA 95616
vv. Department of Environmental, Agricultural, and Occupational Health, College of Public Health, University of Nebraska Medical Center, Omaha, NE 68198
ww. Influenza Division, Centers for Disease Control and Prevention, Atlanta, GA 30329
xx. Armed Forces Health Surveillance Branch, Department of Defense, Silver Spring, MD 20904
yy. Climate Program Office, National Oceanic and Atmospheric Administration, Silver Spring, MD 20910

zz. Leidos supporting the Biomedical Advanced Research and Development Authority, Department of Health and Human Services, Washington, DC 20201
aaa. Bureau of Oceans, International Environmental and Scientific Affairs, U.S. Department of State, Washington, DC 20520
bbb. Office of Science and Technology Policy, The White House, Washington, DC 20502
ccc. BNext, In-Q-Tel, Arlington, VA 22201
ddd. National Center for Medical Intelligence, Defense Intelligence Agency, Fort Detrick, MD 21702

[1]To whom correspondence may be addressed. Email: mjohansson@cdc.gov.

This PDF file includes:

Supplementary Text
Fig. S1 to S5
Tables S1 to S4

Supplementary Text

Forecast Methods

Teams reserved the right to publish their own research and therefore only provided standardized forecasts and brief descriptions of their approaches. The following is a summary of those approaches including citations for those that have been published. Some high-level model characteristics are summarized in Table S1.

Team A. The model is a dynamical two-strain susceptible-exposed-infectious-recovered-susceptible (SEIRS) compartmental model with a multi-life stage model for vector populations. Parameters were derived from the literature and data included dengue case data, precipitation, and minimum and maximum temperatures.

Team B. Forecasts for each target-location were generated from an ensemble of three types of statistical models: Holt-Winters exponential smoothing (time series smoothing of historical dengue at local, seasonal, and long-term scales), multidimensional analogues (on historical dengue data and historical precipitation data), and historical average models (the seasonal distributions of historical cases). Ensemble components were assigned individual weights for each target-location pair based on mean absolute error in predictions over the previous 4 years (1).

Team C. The model used a single strain susceptible-infected-recovered (SIR) model with an ensemble adjustment Kalman filter to sample model parameters consistent with all historical dengue case data available at the time of each prediction (2).

Team D. The model used the K-spectral centroid clustering algorithm to generate normalized clusters of incidence patterns from similar seasons to a sliding window of normalized cases in the current season. The most similar curve was selected and scaled to project mean incidence for the rest of the season. Probabilities for each target were specified based on previous season error for the same targets.

Team E. Two models were used to generate forecasts: a negative binomial generalized linear regression model (including time, recently observed cases, temperature, El Niño, the normalized

difference vegetation index, and temperature-scaled R_0) and a Gaussian process model (including season week, incidence at the end of the previous season, a seasonal sine wave, and an indicator for severe seasons that is estimated for the current season). The Gaussian process model was used for all forecasts with less than 7 seasons of historical data and all early season forecasts, and the negative binomial regression for later season forecasts once more historical data had accumulated (3).

Team F. A generalized linear model was built with numerous variables consisting of different case and environmental variables at different lags. Variables were selected by minimizing collinearity and optimizing fit (on mean absolute error and the Akaike and Bayesian information criteria). For total incidence in San Juan predictions were generated from a second model; a linear regression of cumulative cases reported up to that forecast week in a bootstrap sample of previous seasons on the total number of cases reported.

Team G. A susceptible-infected-recovered (SIR) human and vector compartmental model was fit by iteratively sampling parameters, generating a predictive distribution, evaluating the likelihood on the data, and updating the parameter density. An extremely randomized trees regressor was used to generate independent estimates based on a suite of incidence and weather variables. Estimates from both models were weighted and combined to generate the forecasts.

Team H. Five models were developed to predict subcomponents of dengue risk using historical case data: (1) a seasonal pattern (fitted to a transformed and normalized cosine functions), (2) a local logistic curve (fitted to the current season data), (3) a seasonal autoregressive integrated moving average, (4) a model to predict incidence based on estimated population susceptibility for each season (based on incidence in the previous two seasons), and (5) a model predicting season incidence based on early season incidence. These five model outputs were fitted to historical data with a random forest model to make a single forecast at each time point.

Team I. A 4-strain human compartmental model, with susceptible-exposed-infectious-recovered (SEIR) for unique strain sequence combinations (128 compartments) was coupled with a multi-lifecycle compartmental mosquito model. Parameters were initialized based on literature review and sampled using a genetic algorithm to calibrate the model to the data. The calibrated model was used to generate stochastic simulations for the forecasts.

Team J. Forecasts were made using an ensemble of three models: an empirical Bayes model (using current and historical dengue data) (4), a pinned spline model (using dengue, lagged precipitation, and temperature data), and an empirical prior (using only dengue data from previous seasons). Individual model components were weighted to optimize leave-one-out cross-validation.

Team K. Principal component analysis was used to select four variables from the numerous dengue and climate variables available: lagged minimum temperature, dew point temperature, specific humidity, and precipitation. These variables were used in a multinomial logistic regression to generate forecasts.

Team L. Forecasts were generated by averaging across three models: a neural network informed by a susceptible-infectious-recovered-susceptible (SIRS) compartmental model coupled with a 4-component (egg, immature, susceptible adult, and infectious adult) vector population model, a neural network time series model, and a Bayesian time series model. All models used temperature, precipitation, and historical case data.

Team M. For San Juan, a non-parametric additive autocorrelation model (dimension: 21, time delay: 3, forecasting steps: 52) was fitted using log-transformed data from previous seasons. For Iquitos, a seasonal autoregressive integrated moving average model (SARIMA(3,0,2)(0,1,1)$_{52}$) was used. For both locations, forecasts were made for the entire season and not updated as new data became available within the season.

Team N. The model used ordinary least squares fit to the relationship of cumulative cases up to the current week to each target on historical data and predict incidence for the remainder of the season. Prediction distributions were modified to inflate kurtosis and avoid predictions that were highly unlikely given historical data.

Team O. The model was a non-parametric kernel-density state space reconstruction using log-transformed and smoothed historical dengue data (5). Parameters and lags were estimated independently for each forecast horizon by minimization of the cross-validation mean absolute error of point predictions.

Team P. The model was a Bayesian statistical, time-series regression model including smoothed, lagged dengue case data, lagged climate variables (precipitation, minimum temperature, and relative humidity), and an indicator for serotype switches within the past two years. Cases were modeled as a negative binomial process with an offset for estimated population size.

Climate and environmental data

Daily historical temperature and precipitation observations were made available from the Global Historical Climatology Network. Remotely sensed estimates of precipitation and Normalized Difference Vegetation Index were provided for the areas around both locations from the National Oceanic and Atmospheric Administration (NOAA) Climate Data Records. Temperature, precipitation, dew point, relative humidity, and specific humidity estimates were provided from the National Centers for Environmental Prediction Climate Forecast System Reanalysis. These data sources are maintained and quality controlled by NOAA.

Regression models

We compared logarithmic scores for all team forecasts and the baseline forecasts using Bayesian generalized linear models to estimate the conditional distribution of transformed scores given specific sets of variables potentially related to forecast skill. We first truncated each binned prediction to the range of 0.001 to 0.999 to avoid logarithmic scores of negative infinity or zero. We then calculated the corresponding logarithmic scores and converted those scores to surprisal values ($-\log(p_i)$) by changing the sign. This outcome variable, surprisal, has the advantage of being on the same scale as the logarithmic score but is continuous and positive and therefore suitable to be approximated by a Gamma distribution. The truncation at 0.001 poses one potential complication: for models with many forecasts assigning zero probability to the outcome, the truncation leads to an artificial density at that specific surprisal ($-\log(0.001) \cong 6.9$). However, the Bayesian models treat these observations as uncertain and via Markov Chain Monte Carlo sampling they are distributed across a wider range of scores.

After transformation, we used generalized linear Bayesian regression models to assess the effects of multiple variables (e.g. X_1) on the Gamma-distributed surprisal values (using the identity link to measure effects on the logarithmic score scale):

$$-log(p_i) \sim Gamma(\mu_i, \phi)$$

$\mu_i = \beta_0 + \beta_1 X_{1,i}$,

where ϕ is the dispersion, β_0 and β_1 are regression coefficients. To be consistent with the rest of the manuscript, we reported the estimated effects from the regressions on the logarithmic score scale rather than the surprisal scale.

We fitted regression models with Stan (http://mc-stan.org/) using the stan_glm function in the rstanarm package (http://mc-stan.org/rstanarm/). For each regression, we ran four chains with a burn-in of 500 samples, then collected an additional 1,000 samples and thinned by two to attain 2,000 samples across the four chains. All models were checked for convergence and autocorrelation. To compare regression models we used leave-one-out cross validation with Pareto-smoothed importance sampling to estimate the expected log pointwise predictive density (ELPD) (6).

To assess the relationship between scores and target, location, or season characteristics, we fitted a series of regression models to identify extrinsic factors associated with score variability including: location, target, location-target specific entropy (described below), season, forecast week (week of season), a testing/training season indicator, peak incidence (normalized by location), season incidence (normalized by location), and peak week (centered by location). We calculated target-location entropy ($\sum p \log p$, where p is the bin-specific frequency) as the entropy of each target relative to the target-specific bins over all seasons in the training and testing datasets for each location separately (excluding two seasons in Iquitos with no clear peaks: 2000/2001 and 2011/2012).

First, we fitted a base regression model with covariates for target, forecast week, location and a location-season interaction term to capture variation by season (Table S3). We found that scores varied significantly across at least some components of all of these factors. We then removed the target variable and added two target-related covariates - target-location specific entropy and the number of bins for the probabilistic forecast – finding that the number of bins was associated with the differences in scores by target. We compared the ELPD for this model (-18,182) to the base regression model (-18,184) and found a negligible difference (2.4, standard error (SE): 3.5). We then removed the entropy variable and the location-season interaction terms and added four covariates potentially associated with season-specific scores: an indicator for training versus testing, centered peak week, normalized peak incidence, and normalized season incidence. Scores varied by centered peak week and normalized peak incidence with a slightly lower ELPD (-18,237, difference: -55, SE: 13). We then removed the training season and normalized season incidence variables, keeping only forecast week, location, number of forecast bins, normalized peak incidence, and centered peak week. Compared to the base model, this model had a lower ELPD (-18,234, difference: -50, SE: 15) with a reduced set of variables that could account for key differences in scores. We did not assess additional variables that could explain the between-location difference because with only two locations there would be no resolution between alternative binary effectors.

Next, we used the final model described above to assess differences between forecasting approaches, using variables for mechanistic (if the forecast included at least one mechanistic component), climate (if the forecast used at least one climate variable), and ensemble (if the forecast was based on more than one model) (see Table S1 and above for more model-specific details). Additional important distinctions (e.g. modeling a vector population or using serotype data) were not assessed because not enough forecasts incorporated those approaches to enable comparison. For example, only two forecasts used serotype data and each used it in a different

way so any comparison would be confounded by many other possible distinctions (e.g. which climate data were used).

Finally, we compared calibration (see Fig. S4 for the specific metric) by forecasting approach with the same three characteristics (mechanistic, climate, and ensemble) while controlling for target and location (Table S4).

References

1. Buczak AL*, et al.* (2018) Ensemble method for dengue prediction. *PLoS One* 13(1):e0189988.
2. Yamana TK, Kandula S, & Shaman J (2016) Superensemble forecasts of dengue outbreaks. *J. Royal Soc. Interface* 13(123):20160410.
3. Johnson LR*, et al.* (2018) Phenomenological forecasting of disease incidence using heteroskedastic Gaussian processes: A dengue case study. *Ann. Appl. Stat.* 12(1):27-66.
4. Brooks LC, Farrow DC, Hyun S, Tibshirani RJ, & Rosenfeld R (2015) Flexible modeling of epidemics with an empirical Bayes framework. *PLoS Comput. Biol.* 11(8):e1004382.
5. Ray EL, Sakrejda K, Lauer SA, Johansson MA, & Reich NG (2017) Infectious disease prediction with kernel conditional density estimation. *Stat. Med.* 36(30):4908-4929.
6. Vehtari A, Gelman A, & Gabry J (2017) Practical Bayesian model evaluation using leave-one-out cross-validation and WAIC. *Stat. Comput.* 27(5):1413-1432.

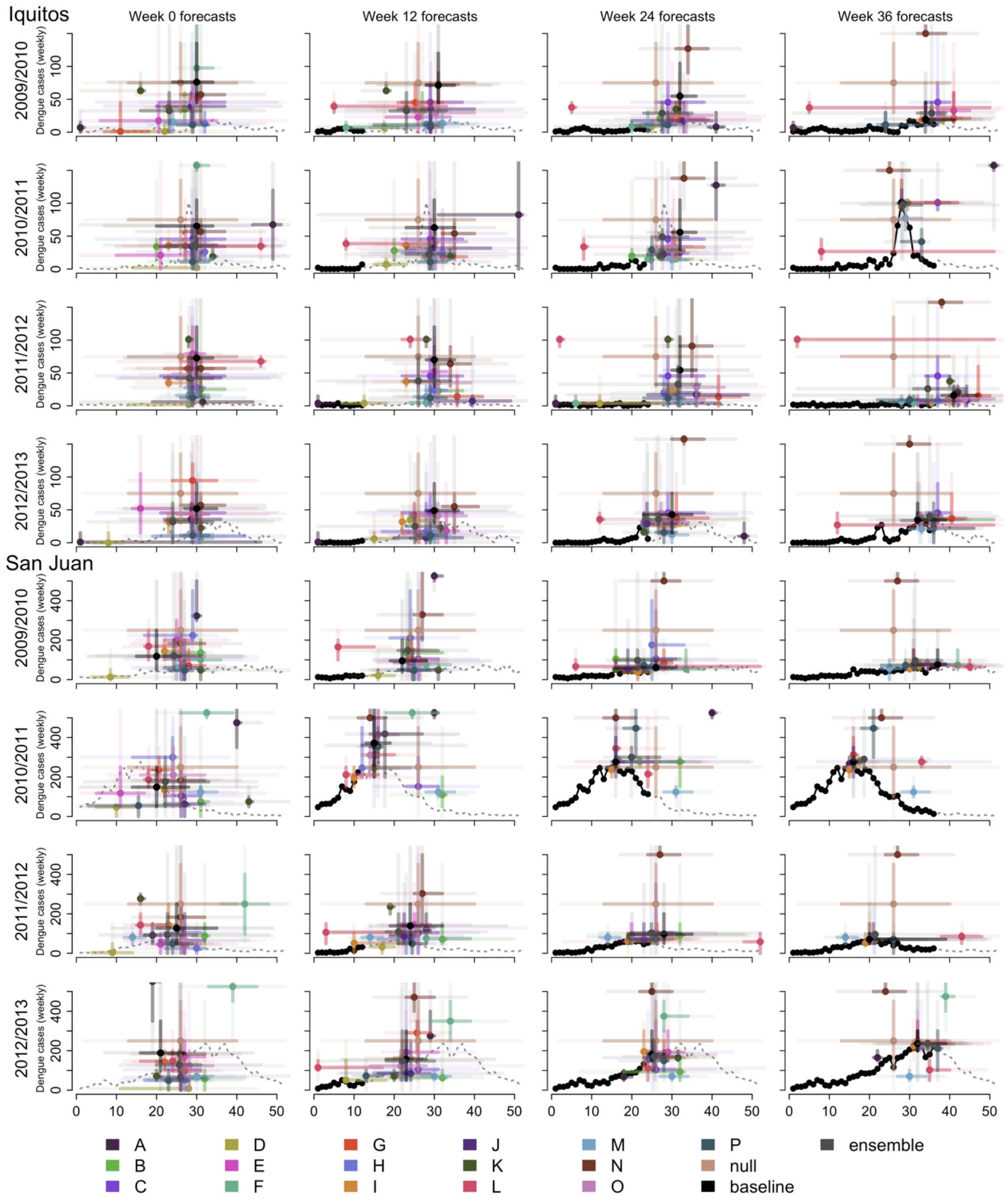

Fig. S1. Peak week and peak incidence forecasts at weeks 0, 12, 24, and 36 for all testing seasons. The solid black lines indicate the most recent dengue data that were available to teams to inform these forecasts and the dashed line indicates the data that became available later in the season. The colored points represent point estimates for each team while the bars represent 50% and 95% prediction intervals (dark and light, respectively). The colors match the legend in Fig. 2 and the labels in Fig. S2. Corresponding forecasts for seasonal incidence are shown in Fig. S2.

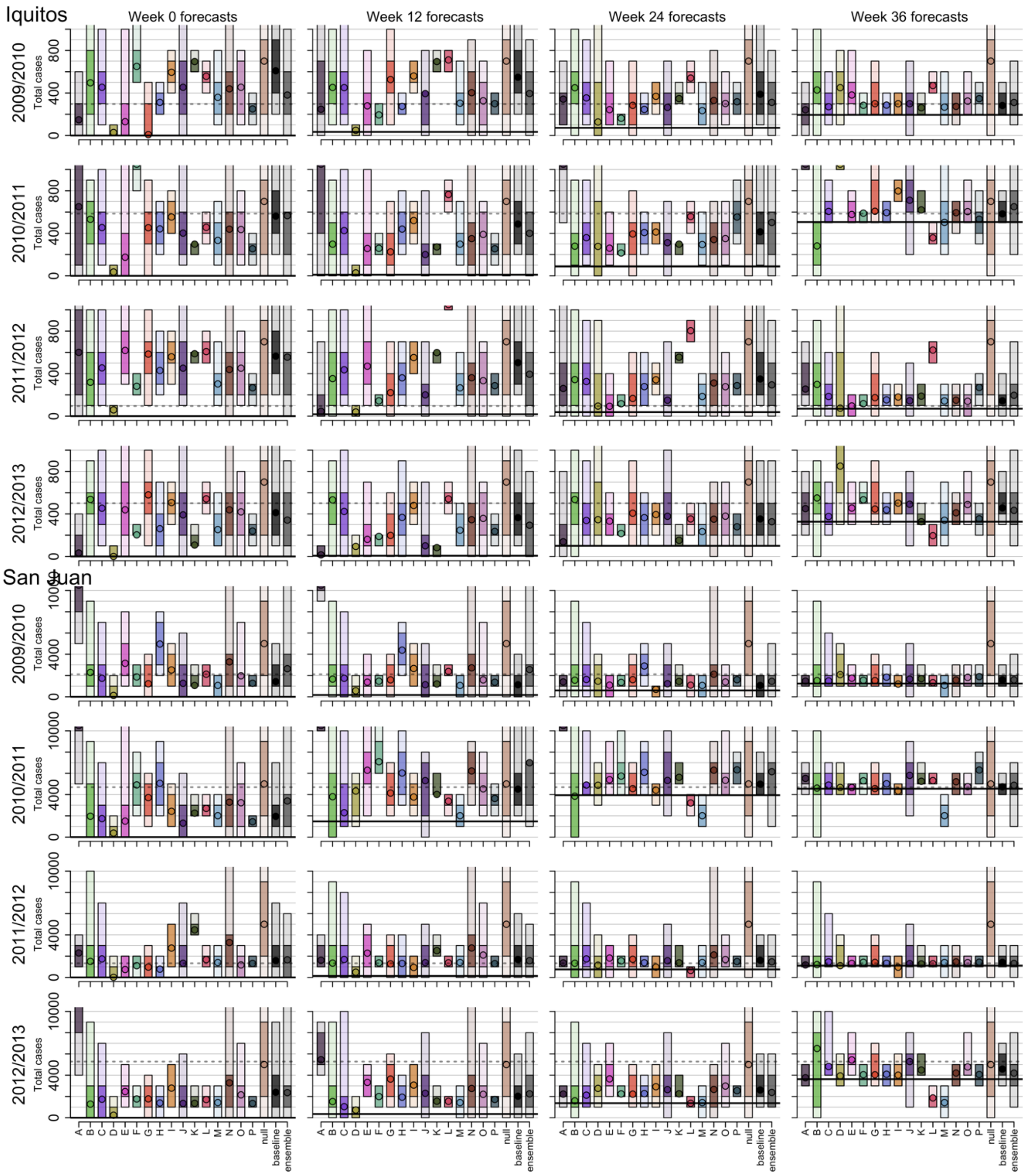

Fig. S2. Total seasonal incidence forecasts at weeks 0, 12, 24, and 36 for all testing seasons. The solid black lines indicate the number of cases reported up to week 12 or week 24 and the dashed lines indicate the total at the end of the season. Grey horizontal lines indicate the bins for each forecast. Points are the point estimates and shading represents the 50% and 95% prediction intervals (dark and light, respectively). Open bars at the top indicate that the 95% interval includes the highest bin: greater than 1,000 or 10,000 cases, for Iquitos or San Juan, respectively.

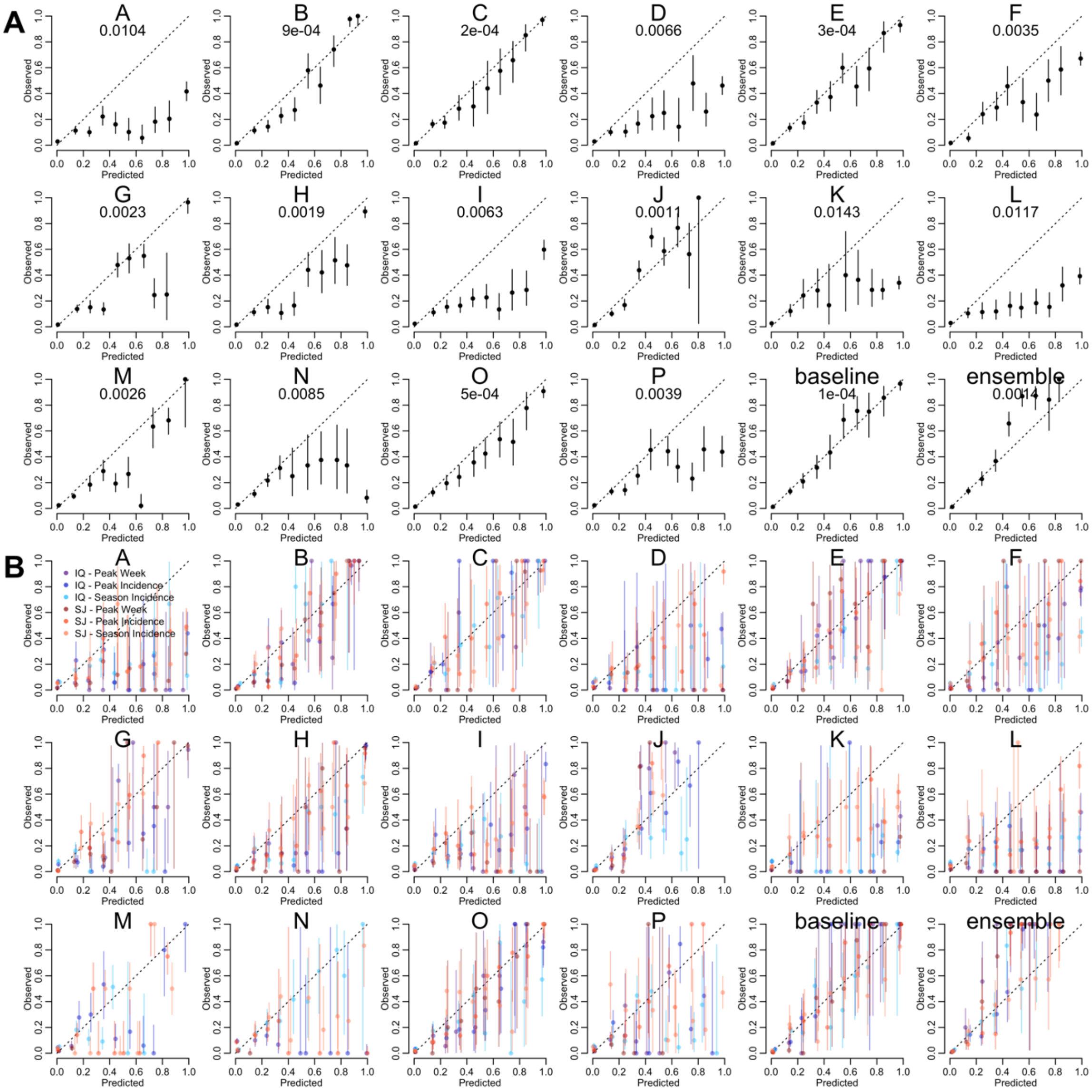

Fig. S3. Calibration of forecasts by team. Team-specific calibration for binned forecasts overall (**A**) and by target-location (**B**). Each point indicates the average forecast probability within a given bin (e.g. between 0.2 and 0.3) and the frequency of the observed outcome for those forecasts. The vertical line is the confidence interval for that mean (a large interval indicates fewer forecasts in that bin). The dashed diagonal line indicates ideal calibration and the number under the team name is a calibration metric indicating distance from this line (lower calibration is closer to zero distance and therefore better). Calibration was calculated as the mean weighted squared difference $\frac{1}{N}\sum \mathrm{n}_k(\bar{\mathrm{p}}_k - \bar{\mathrm{o}}_k)^2$, where N is the total number of forecasts, n_k is the number of forecasts in bin k with average probability $\bar{\mathrm{p}}_k$, and $\bar{\mathrm{o}}_k$ is the frequency of those forecasts being correct. Panel B shows the calibration for each target-location pair.

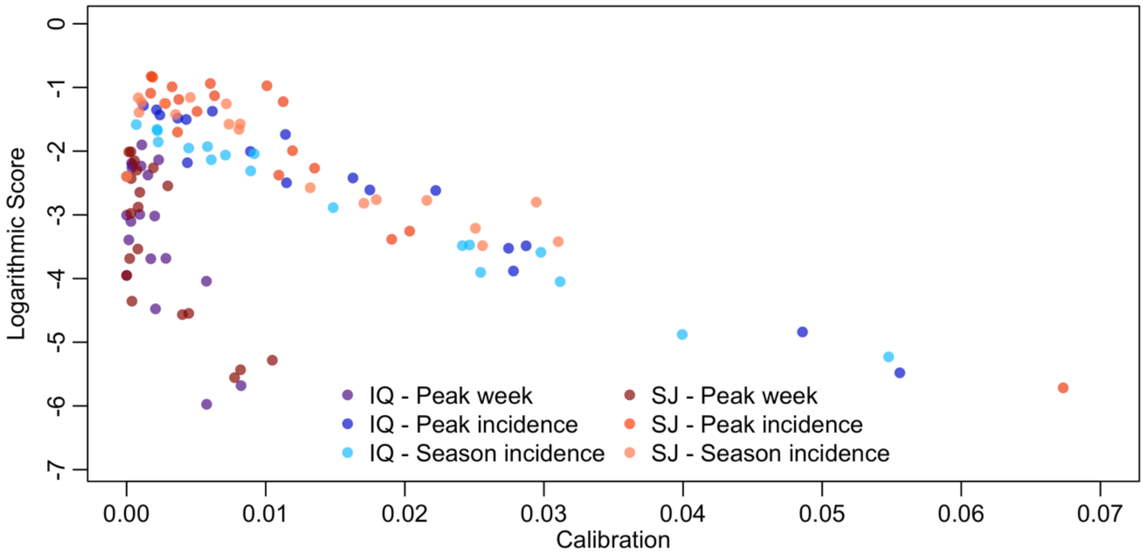

Fig. S4. Calibration compared to logarithmic score by team and target. Calibration was calculated as described in Fig. S3. Better calibration (lower) was general associated with better logarithmic scores (higher) for all target-location pairs (Iquitos-Peak week R^2: 0.55, Iquitos-Peak incidence R^2: 0.91, Iquitos-Season incidence R^2: 0.95, San Juan-Peak week R^2: 0.59, San Juan-Peak incidence R^2: 0.79, San Juan-Season incidence R^2: 0.78). Despite lower logarithmic scores, peak week calibration is generally better because the greater number of bins (52 vs. 11) leads to more low or no probability predictions for outcomes that did not happen, which are therefore well calibrated.

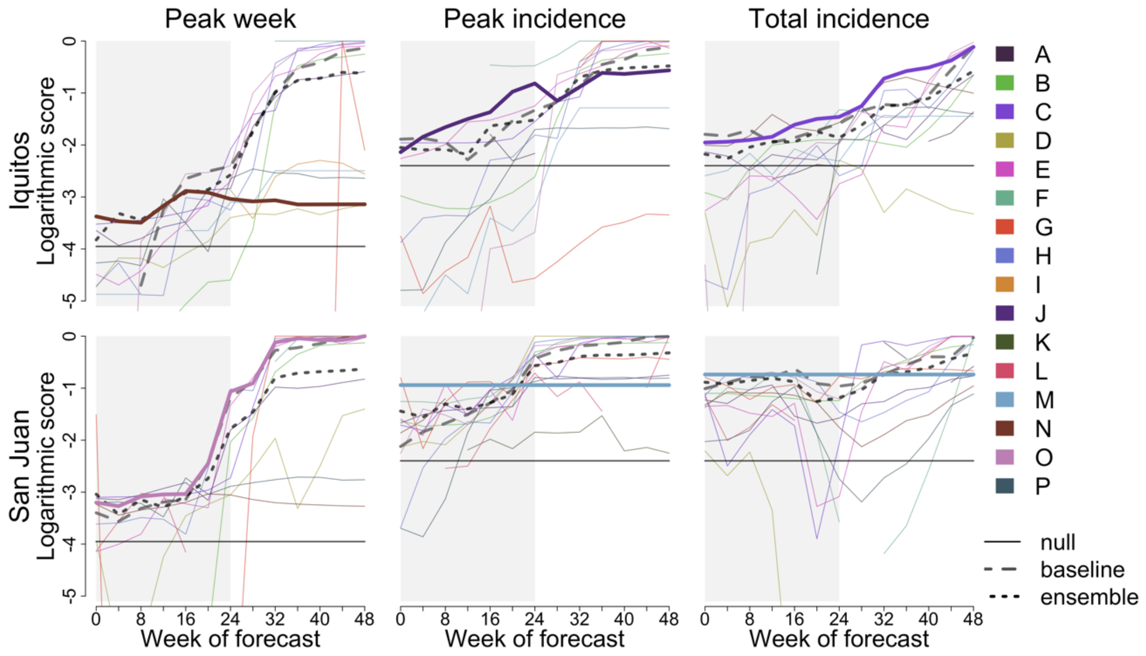

Fig. S5. Forecast skill by team, forecast week, and target in the training seasons (2005/2006 to 2008/2009). Solid colored lines represent the scores of individual teams averaged across all testing seasons for the respective forecast week, target, and location. For each target, the top forecast for the first 24 weeks (shaded) is indicated in bold (highest average early season score). The solid black line indicates the null model (equal probability assigned to all possible outcomes), the dashed grey line the baseline model, and the dotted black line the ensemble model. Forecasts with logarithmic scores of less than -5 are not shown. Breaks in lines indicate a score of negative infinity in at least one of the testing seasons.

Table S1. Model characteristics and forecasting scores for Weeks 0-24 in the testing seasons (2009/2010 to 2012/2013). The highest score for each target is indicated in bold, with both the top team and the baseline indicated if the baseline outperformed all teams.

	**Model Characteristics**				**San Juan (logarithmic scores)**			**Iquitos (logarithmic scores)**		
**Team**	Mech.‡	Ensemble	Climate	Serotype	Peak Incidence	Peak Week	Season Incidence	Peak Incidence	Peak Week	Season Incidence
**A**	Yes	No	Yes	No	-4.62†	-6.02†	-4.79†	-3.08†	-6.07†	-3.03†
**B**	No	Yes	Yes	No	-2.57	-4.24	-2.04*	**-1.85***	-6.38	**-2.03***
**C**	Yes	Yes	No	No	-2.12*	-4.14	-2.13*	-2.08*	-3.36*	-2.47
**D**	No	No	No	No	-2.62	-6.45†	-5.07	-4.76	-5.83	-5.43
**E**	No	Yes	Yes	No	-1.43*	**-3.70***	-2.81	-2.54	-3.29*	-3.14
**F**	No	Yes	Yes	No	-2.99†	-3.98†	-3.98†	-5.45†	-4.66†	-6.71†
**G**	Yes	Yes	Yes	No	**-1.23***	-4.88	-1.992*	-2.18*	-3.36*	-2.49
**H**	No	Yes	No	No	-2.60	-5.37	-2.64	-3.07	-3.45*	-2.54
**I**	Yes	No	Yes	No	-2.95†	-6.03†	-3.32†	-4.18†	-3.91*†	-4.02†
**J**	No	Yes	Yes	No	-1.74*	-4.20	**-1.986***	-2.27*	-3.61*	-2.66
**K**	No	No	Yes	No	-4.49†	-6.17†	-4.38†	-6.68†	-6.91†	-6.91†
**L**	Yes	Yes	Yes	No	-3.30†	-5.45†	-4.19†	-5.29†	-4.05†	-4.61†
**M**	No	No	No	No	-5.17	-5.75†	-7.66	-5.78	-2.98*	-3.50
**N**	No	No	No	No	-4.98†	-4.06	-2.18*	-3.88†	**-2.96***	-2.28*
**O**	No	No	No	No	-2.66	-3.90*	-2.84	-5.24	-4.35	-3.61
**P**	No	No	Yes	Yes	-2.63†	-5.36†	-4.55†	-2.89†	-3.08*	-4.81†
**null**	NA	No	No	No	-2.40	-3.95	-2.40	-2.40	-3.95	-2.40
**baseline**	No	No	Yes	No	-1.43*	**-3.47***	-2.15*	-2.88	**-2.55***	-3.62†
**ensemble**	Yes	Yes	Yes	Yes	-1.68*	-3.60*	-2.13*	-2.14*	-3.10*	-2.09*

‡Yes if the model included any mechanistic component.
†Forecasts with zero probability assigned to at least one observed outcome. Those individual forecast probabilities were changed to 0.001 to calculate the average.
*Forecasts with scores higher than the null model.

Table S2. Forecasting scores for Weeks 0-24 in the training seasons (2005/2006 to 2008/2009). The highest score for each target is indicated in bold, with both the top team and the baseline indicated if the baseline outperformed all teams.

	**San Juan (logarithmic scores)**			**Iquitos (logarithmic scores)**		
**Team**	Peak Incidence	Peak Week	Season Incidence	Peak Incidence	Peak Week	Season Incidence
**A**	-4.74†	-6.36†	-5.04†	-2.95†	-6.11†	-3.25†
**B**	-1.43*	-5.11	-1.17*	-3.02	-5.18	-2.31*
**C**	-1.22*	-3.01*	-2.09*	-1.89*	-3.20*	**-1.74***
**D**	-1.44*	-4.27	-4.83	-8.34†	-4.11	-3.62
**E**	-1.28*	-3.28*	-1.91*	-1.73*	-3.82*	-2.95
**F**	-1.06*	-5.31†	-1.47*	-1.45*†	-5.29†	-2.43†
**G**	-1.10*	-9.48	-0.99*	-4.23	-22.26	-12.76
**H**	-1.81*	-3.08*	-0.93*	-2.90	-4.01	-3.17
**I**	-5.20†	-4.12†	-2.12*†	-3.80†	-5.86†	-2.91†
**J**	-1.36*	-2.86*	-1.33*	**-1.47***	-3.45*	-2.22*
**K**	-2.61†	-6.91†	-2.18*†	-4.97†	-5.18†	-5.80†
**L**	-2.21*†	-4.15†	-2.60†	-3.82†	-6.72†	-4.70†
**M**	**-0.94***	-3.54*†	**-0.74***	-4.30	-4.25†	-2.35*
**N**	-4.41†	-3.23*	-1.25*	-4.63†	**-3.19***	-1.75*
**O**	-1.31*	**-2.74***	-0.91*	-6.11	-5.86	-3.89
**P**	-2.21*	-3.11*	-1.81*	-3.42	-3.72*	-4.16†
**null**	-2.40	-3.95	-2.40	-2.40	-3.95	-2.40
**baseline**	-1.41*	-2.89*	-0.85*	-1.83*	-3.41*†	-1.78*
**ensemble**	-1.24*	-2.93*	-0.97*	-1.88*	-3.16*	-1.99*

‡Yes if the model included any mechanistic component.
†Forecasts with zero probability assigned to at least one observed outcome. Those individual forecast probabilities were changed to 0.001 to calculate the average.
*Forecasts with scores higher than the null model.

Table S3. Regression models comparing logarithmic scores by target, location, season, and modeling-approach.

	Variable	Mean	Lower 95% CI	Upper 95% CI	ELPD*	SE
Base					-18184	171
	**Forecast week**	**0.0408**	**0.0371**	**0.0446**		
	**Iquitos-2006/2007**	**0.906**	**0.602**	**1.22**		
	Iquitos-2007/2008	-0.14	-0.515	0.226		
	Iquitos-2008/2009	-0.242	-0.624	0.139		
	Iquitos-2009/2010	0.256	-0.084	0.607		
	Iquitos-2010/2011	-0.27	-0.653	0.126		
	**Iquitos-2011/2012**	**-0.428**	**-0.863**	**-0.00202**		
	Iquitos-2012/2013	0.132	-0.237	0.509		
	**San Juan**	**0.91**	**0.628**	**1.21**		
	**San Juan-2006/2007**	**0.301**	**0.114**	**0.471**		
	**San Juan-2007/2008**	**-0.386**	**-0.65**	**-0.136**		
	**San Juan-2008/2009**	**-0.271**	**-0.519**	**-0.0424**		
	**San Juan-2009/2010**	**-0.927**	**-1.28**	**-0.619**		
	**San Juan-2010/2011**	**-0.649**	**-0.957**	**-0.385**		
	**San Juan-2011/2012**	**0.452**	**0.278**	**0.626**		
	**San Juan-2012/2013**	**-1.49**	**-1.87**	**-1.13**		
	**Target: Peak Week**	**-1.15**	**-1.32**	**-0.994**		
	**Target: Season Incidence**	**-0.0489**	**-0.156**	**0.0544**		
Target variables					-18182	170
	**Forecast week**	**0.0406**	**0.0369**	**0.0444**		
	**Iquitos-2006/2007**	**0.895**	**0.607**	**1.19**		
	Iquitos-2007/2008	-0.135	-0.515	0.218		
	Iquitos-2008/2009	-0.247	-0.642	0.119		
	Iquitos-2009/2010	0.247	-0.114	0.566		
	Iquitos-2010/2011	-0.275	-0.671	0.0976		
	**Iquitos-2011/2012**	**-0.47**	**-0.898**	**-0.0385**		
	Iquitos-2012/2013	0.107	-0.246	0.447		
	**San Juan**	**0.921**	**0.646**	**1.2**		
	**San Juan-2006/2007**	**0.294**	**0.112**	**0.479**		
	**San Juan-2007/2008**	**-0.369**	**-0.639**	**-0.122**		
	**San Juan-2008/2009**	**-0.291**	**-0.532**	**-0.0493**		
	**San Juan-2009/2010**	**-0.959**	**-1.31**	**-0.647**		
	**San Juan-2010/2011**	**-0.648**	**-0.932**	**-0.378**		
	**San Juan-2011/2012**	**0.483**	**0.318**	**0.649**		
	**San Juan-2012/2013**	**-1.5**	**-1.88**	**-1.15**		
	**Number of bins**	**-0.0268**	**-0.0309**	**-0.023**		
	Target entropy	-0.116	-0.316	0.103		
Target & season variables					-18237	171
	**Forecast week**	**0.0424**	**0.0385**	**0.0464**		
	**San Juan**	**0.664**	**0.554**	**0.777**		
	**Number of bins**	**-0.0257**	**-0.0295**	**-0.022**		
	**Centered peak week**	**-0.0488**	**-0.0575**	**-0.0402**		
	**Normalized peak incidence**	**-0.405**	**-0.549**	**-0.26**		
	Normalized season incidence	-0.0345	-0.163	0.0968		
	Training season	-0.058	-0.169	0.0565		
Reduced					-18234	171
	**Forecast week**	**0.0425**	**0.0388**	**0.0464**		
	**San Juan**	**0.652**	**0.542**	**0.762**		
	**Number of bins**	**-0.0257**	**-0.0293**	**-0.0221**		

	**Centered peak week**	**-0.0479**	**-0.0565**	**-0.0397**		
	**Normalized peak incidence**	**-0.429**	**-0.489**	**-0.371**		
Model type					-18024	168
	**Forecast week**	**0.0438**	**0.0397**	**0.0481**		
	**San Juan**	**0.409**	**0.303**	**0.52**		
	**Number of bins**	**-0.0155**	**-0.0196**	**-0.0115**		
	**Centered peak week**	**-0.0369**	**-0.0447**	**-0.0298**		
	**Normalized peak incidence**	**-0.186**	**-0.239**	**-0.143**		
	**Climate**	**-0.136**	**-0.185**	**-0.0942**		
	**Ensemble**	**1.02**	**0.909**	**1.13**		
	**Mechanistic**	**-0.645**	**-0.802**	**-0.492**		

*ELPD: Leave-One-Out Expected Log Pointwise predictive Density

Table S4. Regression model comparing calibration by target, location, and modeling-approach.

Variable	Mean	Lower 95% CI	Upper 95% CI
Climate	0.00120	-0.00046	0.00307
Ensemble	-0.00096	-0.00335	0.00070
Mechanistic	0.00196	-0.00060	0.00604
San Juan	0.00005	-0.00142	0.00158
**Target: Peak Week**	**-0.01230**	**-0.01900**	**-0.00737**
Target: Season Incidence	-0.00067	-0.00749	0.00990
